# *Annona cherimola* Mill. Leaf Extracts Affect Melanoma Cells Growth and Progression

**DOI:** 10.3390/foods11162420

**Published:** 2022-08-11

**Authors:** Domenico Iacopetta, Alessia Fazio, Chiara La Torre, Alexia Barbarossa, Jessica Ceramella, Fabrizio Francomano, Carmela Saturnino, Hussein El-Kashef, Stefano Alcaro, Maria Stefania Sinicropi

**Affiliations:** 1 Department of Pharmacy, Health and Nutritional Sciences, University of Calabria, 87036 Arcavacata, di Rende, Italy; 2 Department of Pharmacy-Drug Sciences, University of Bari “Aldo Moro”, 70126 Bari, Italy; 3 Department of Science, University of Basilicata, 85100 Potenza, Italy; 4 Chemistry Department, Faculty of Science, Assiut University, Assiut 17516, Egypt; 5 Dipartimento di Scienze della Salute, Università “Magna Græcia” di Catanzaro, Viale Europa, 88100 Catanzaro, Italy; 6 Net4Science SRL, Academic Spinoff, Università “Magna Græcia” di Catanzaro, Viale Europa, 88100 Catanzaro, Italy; 7 Associazione CRISEA-Centro di Ricerca e Servizi Avanzati per l’Innovazione Rurale, Belcastro, 88055 Catanzaro, Italy

**Keywords:** *Annona cherimola*, melanoma, tubulin, actin, epithelial–mesenchymal transition markers

## Abstract

Cancer represents one of the major causes of mortality worldwide; indeed, 19.3 million new cases and almost 10.0 million deaths were estimated last year. Among the different type of cancers, malignant melanoma represents the most aggressive and deadly skin cancer. Unfortunately, the long-term efficacy of melanoma treatments is limited by the lack of clinical efficacy, onset of side effects and resistance. The latter is a major obstacle for the success of the melanoma therapy; thus, the exploration of new potent and safer anticancer agents is of great importance. Recently, numerous plant species, used for therapeutic purposes and containing various non-toxic nutraceuticals have been widely studied. Herein, we investigated the antioxidant and anticancer properties on melanoma cells of the ethanolic, methanolic and aqueous *Annona cherimola* leaf extracts (ACE, ACM and ACW, respectively). The ethanolic extract showed higher anticancer activity, mostly against the malignant A2058 melanoma cell line (IC_50_ = 5.6 ± 0.8 ng/mL), together with a very low activity on the normal cells. It blocks the melanoma cells migration process, and induces a clear disorganization of cytoskeleton, triggering cell apoptosis. Finally, some bioactive compounds were identified in the studied extracts.

## 1. Introduction

Since ancient times, several plants have aroused great interest in folk medicine for the treatment of several diseases [1], while in the present years, their bioactive compounds have stimulated interesting research in the pharmaceutical, nutraceutical and cosmetic fields.

The study of the anticancer properties of vegetable-derived compounds falls in the medicinal chemistry and nutraceuticals areas, offering alternative strategies to prevent or treat several diseases, and among all plant organs, leaves are the largest accumulators of bioactive compounds, stored as secondary metabolites [2,3,4]. The *Annona* genus, belonging to the *Annonaceae* family, includes more than 119 species, and among them, *Annona cherimola* Mill. (or *Annona tripetala* Ait. or Cherimoya) is a subtropical fruit, native to Colombia, Ecuador, Peru, Bolivia and Chile [5], spreading through cultivation to the Andes and Central America. It is cultivated, as well, on the coast of Granada–Malaga (Spain) and the south of Italy (Calabria and Sicily). Cherimoya is a mostly evergreen, low-branched spreading tree or shrub, which can reach a height between 5 and 9 m. The leaves, deciduous and semi-deciduous, are variable in shape (ovate, ovate-lanceolate, obovate or elliptical) and dimension (12–20 × 8 cm); they are brownish, alternate leaves, slightly hairy on the upper surface and smooth on the lower one [6]. The leaves are traditionally used in Mexico, South America, India and the Azores to prepare decoctions for treating diarrhea, intestinal worms, respiratory diseases, hyperlipidemia, hyperglycemia, anxiety, convulsions and agitation [7]. Some of the traditional uses of the leaves were legitimated, successively, by scientific studies; their extracts were found to be an excellent source of bioactive compounds, such as flavonoids, tannins, alkaloids, phytosterols and terpenoids [5].

Due to the interesting phytochemical profile, the pharmacological potential of Cherimoya leaves has been explored [8] and the rutine-rich decoction of *A. cherimola* leaves has been used to treat hypercholesterolemia, because of the inhibition of the HMG-CoA reductase activity [9,10].

The hexane extract of the leaves of *A. cherimola* produced anxiolytic-like actions in mice, the effect antagonized by picrotoxin, a GABA-gated chloride ion channel blocker. The proven presence of several compounds such as β-caryophyllene, β-selinene, α-cubebene and linalool could explain some of the extract effects [11,12]. The ethanolic extract inhibited the replication of HSV-2 (herpes simplex virus type 2) in HEp-2 cells, with a good therapeutic index, showing potentiality as an antiviral [13].

The methanolic extract of the leaves from Mexican *A. cherimola* exhibited in vitro antimicrobial and antibiotic activities against *Helicobacter pylori* [14], *Entamoeba histolytica* and *Giardia lamblia*, respectively [15]. Moreover, the antibacterial activity of the essential oil from *A. cherimola* leaves against *Campylobacter jejuni* was recently reported by Valarezo et al. [16].

Other studies on ethanolic leaf extracts revealed anti-proliferative and pro-apoptotic effects on the chemo-resistant MDA-MB-231 breast cancer cells [17] and on acute myeloid leukemia cell lines [18].

Herein, we reported the phenolic profile, flavonoid content and antioxidant capacity of the methanolic, ethanolic and aqueous extracts of leaves from *A. cherimola* Mill. (ACM, ACE and ACW, respectively) grown in the Tyrrhenian coast of Calabria, in Southern Italy. We also investigated the anticancer activity of these extracts against two human melanoma cell lines, A2058 and Sk-Mel-28, and the lack of cytotoxicity against the embryonal mouse fibroblasts 3T3-L1. We observed the apoptosis induction and the inhibition of the metastatic potential of A2058 cells treated with our extracts, suggesting that the reduction in melanoma cells migration is associated with the modulation of some of the main proteins involved in the epithelial-to-mesenchymal transition (EMT) process, namely E- and N-cadherin and vimentin, as well as with the perturbation of tubulin and actin cytoskeleton. Finally, the reduction in intracellular VEGF production in A2058 cells and the ROS scavenging activity in human embryonic kidney Hek-293 cells was also evidenced. Overall, our outcomes suggest new insight regarding the mechanisms of action of *A. cherimola* leaf extracts, a valuable source of compounds with a high potential in melanoma prevention and/or treatment.

## 2. Materials and Methods

### 2.1. Chemicals and Reagents

All solvents of analytical grade, water (HPLC grade), acetonitrile (HPLC grade) and sodium carbonate were purchased from Carlo Erba Reagent (Milan, Italy).

Folin-Ciocâlteu reagent, 2,2-diphenyl-1-picrylhydrazyl radical, 2,2′-azino-bis (3-ethylbenzothiazoline-6-sulphonic acid) diammonium salt, Trolox (6-hydroxy-2,5,7,8-tetramethylchroman-2-carboxylic acid), aluminium chloride, gallic acid, quercetin, ferulic acid, chlorogenic acid, 3-(4,5-dimethylthiazol- 2-yl)-2,5-diphenyltetrazolium bromide reagent (MTT), menadione, 2′-7′-Dichlorofluorescein diacetate (DCF-DA) and fetal bovine serum (FBS) were purchased from Sigma Aldrich S.r.l., Milan (MI), Italy, Syringe filters in PVDF, pore size 0.45 µm (Millipore) were from SER.DIA s.r.l., Reggio Calabria (RC), Italy

### 2.2. Plant Material

The leaves of *A. cherimola* Mill., belonging to *Autoctona Calabrese* cultivar, were picked up in October 2018 from trees grown in a hilly area of Gallico (latitude 38°09′50.9″ N and 15°39′06.88″ E), a district of the municipality of Reggio Calabria (Southern Italy, Calabria), and authenticated by the Botanical Garden of University of Calabria. Specimens were deposited with voucher number 26,235. The leaves were washed and cleaned of any impurities, preserved under nitrogen, frozen at −20 °C and then freeze dried (Lyophilizer Telstar LyoQuest, Barcelona, Spain). After all the dried leaves were manually ground in a mortar with a pistil, sieved through a 60-mesh (260 µm) screen and stored at −20 °C before the extractions. All extractions were performed in triplicate.

### 2.3. Preparation of the Leaf Extracts

The extraction of polar compounds from *A. cherimola* leaves was carried out using solvents with growing polarity: ethanol, methanol and water. The extraction with each solvent was conducted in triplicate on 3× *g* of dry leaf powder. Briefly, leaf powder (3× *g*) was added by 30 mL of each solvent (10 mL g^−1^) in capped plastic tubes and left under magnetic stirring for 2 h at room temperature. The supernatant was separated from the solid residue by centrifugation (5000 rpm for 15 min) and the resulting solid was extracted two more times with the solvent using the same procedure. The filtrates were combined and filtered by sintered glass Buchner funnel (4 µm pore size) in a weighed flask. The organic solvent from the three extracts (ethanol, methanol and water) was separately removed under reduced pression. The recovery of leaf extracts was expressed as milligrams per gram of dried leaf powder. Data were reported as the average value of three replicates ± standard deviation.

### 2.4. Determination of Total Phenolic Content

Total phenolic content of the three extracts was determined by the Folin–Ciocâlteu colorimetric method [19]. Briefly, 100 µL of extract solution (1.0 mg mL^−1^ DMSO) was dissolved in 1.1 mL of distilled water and added 1 mL of diluted (1:10) Folin–Ciocâlteu reagent. After allowing the mixture to incubate for 5 min, 800 µL of 10% Na_2_CO_3_ was added. Incubation of the mixture was then carried out for 2 h at room temperature, in the dark, before measuring the absorbance at 765 nm by a UV–vis spectrophotometer (model V-550, Jasco, Europe), using appropriate blanks for background subtraction. The TPC in each extract was determined by constructing calibration curves of gallic acid within the 0.01 to 0.5 mg mL^−1^ range concentration and expressed as micrograms of gallic acid equivalents (µg GAE) per gram of dried extract. Data were expressed as the means of three assays on each extract ± standard deviation.

### 2.5. Determination of Total Flavonoid Content

The total flavonoid content (TFC) was determined using a colorimetric assay method [20]. Briefly, 300 µL of extract solution (1 mg mL^−1^ DMSO) dissolved in 900 µL of methanol was mixed with 60 µL of sodium acetate solution (1 M) and 60 µL of 10% aluminium chloride solution and 1.68 mL of distilled water. The mixture was left to incubate in the dark for 30 min under stirring, at room temperature. The absorbance was read at 420 nm by a UV–vis spectrophotometer against a blank prepared by replacing the extract with DMSO. All the determinations were performed in three different replicates and the results were expressed as the mean values ± standard deviation. The TFC was calculated from a standard calibration curve 0.01–0.5 mg mL^−1^ curve for quercetin and expressed as micrograms of quercetin equivalents (µg QE) per gram of dried extract.

### 2.6. Radical Scavenging Activity

Antioxidant activity of the ethanolic, methanolic and aqueous extracts from leaves was determined by DPPH and ABTS assays.

#### 2.6.1. Free Radical Scavenging Activity (DPPH)

The DPPH radical scavenging capacity assay was performed as described by Malacaria et al. [21].

Four solutions at different concentrations (1.0, 0.5, 0.1 and 0.01 mg mL^−1^ of DMSO) were prepared for each extract and tested to obtain the DPPH inhibition percentage (%I_DPPH_), expressed as mean value of the three results ± standard deviation. The inhibition percentages of each extract were used to determine EC_50_ values.

The radical scavenging activity was expressed as Trolox equivalent antioxidant capacity (TEAC), and a standard curve was created using 0.001–1 mg mL^−1^ Trolox solutions.

#### 2.6.2. ABTS Radical Cation Scavenging Activity

This assay is based on the scavenging of 2, 2′-azino-bis (3-ethylbenzothiazoline-6-sulphonate) radical cation (ABTS•^+^).

ABTS•^+^ radical cation was prepared by mixing 7 mM ABTS solution and 2.45 mM potassium persulfate and allowing the mixture to stand in darkness at room temperature for about 12–16 h. Then, ABTS solution was diluted in ethanol (1:25) until the absorbance reached the value of 0.700–0.730 at 734 nm.

For each extract, four standard solutions were prepared by using DMSO as solvent (1.0, 0.5, 0.1 and 0.01 mg mL^−1^), and then they were tested to determine their ABTS inhibition percentages (%IABTS) as described by Iacopetta et al. [22]. All the determinations were performed in three different replicates and the %I_ABTS_ values of each sample were expressed as the average of the three results ± standard deviation. The inhibition percentages of each extract were used to determine EC_50_ values.

The ABTS radical cation scavenging activities were also expressed as expressed as milligrams of Trolox equivalents per gram of dry extract (TEs/g extract).

### 2.7. Identification and Quantification of Phenolic Compounds by High-Performance Liquid Chromatography–Diode Array Detector

Qualitative and quantitative phenolic profile was determined by HPLC-DAD (Shimadzu, Kyoto, Japan) equipped with Auto Sampler (SIL-20A), pumps (LC-20AD), oven (CTO-10AS vp), diode array detector (SPD-M20A) and a system controller CMB-20A.

The phenolic compounds were identified in the extracts by both standard addition and comparison of their retention times, and UV spectra with those of commercial standards in the same chromatographic and with those previously reported in the literature [23]. Chromatographic analyses were conducted using a C18 column (Adamas^®^ 250 mm × 4.6 mmID, 5 µm particle size) (SupaChrom Srl, Rho, MI, Italy), thermostated at 25 °C (±1 °C). Mobile phase consisted of water containing 0.2% of formic acid (A) and acetonitrile (B). All the analyses were performed in gradient elution mode as follows: 0–10 min 17% of B, 10–20 min 20% of B, 20–30 min 55% of B, 30–40 min 58% of B, 40–50 min 58% of B, 50–60 min 70% of B, 60–70 min 20% of B and 70–80 min the gradient return of 17% of B. The operating conditions of each analysis were the following: flow rate of 0.6 mL min^−1^, injection volume of 20 µL, and wavelengths of 271 nm for gallic acid, 276 nm for *p*-coumaric acid, 280 nm for ferulic acid, 327 nm for chlorogenic acid and 365 nm for quercetin.

The qualitative analysis was performed by comparing retention times of compounds in the chromatograms of the extracts with the standard ones and by analyzing each extract enriched with the standards one by one. The individual polyphenolic compounds were quantified by the external standard method using the calibration curves of the following commercial standards (1–500 µg mL^−1^) and by plotting HPLC peak areas vs. concentrations. Vanillic acid, ellagic acid, quercetin, p-coumaric acid, ferulic acid, chlorogenic acid and gallic acid were detected at appropriate wavelengths (280, 254, 365, 271, 325, 327 and 276 nm, respectively).

The Annona extracts (ethanol, methanol and water) were analyzed at a concentration of 1 mg mL^−1^ after filtering samples through a 0.45 µm membrane filter (Millipore).

### 2.8. Biology

#### 2.8.1. Cell Culture

The cell lines used in this work were purchased from American Type Culture Collection (ATCC, Manassas, VA, USA). A2058 epithelial melanoma cells were cultured in Dulbecco’s modified Eagle’s medium (DMEM) low glucose (1 g/L) supplemented with 1% L-glutamine, 100 U/mL penicillin/streptomycin and 20% Fetal Bovine Serum (FBS). Sk-Mel-28 malignant melanoma cells and human embryonic kidney Hek-293 were maintained in DMEM high glucose (4.5 g/L) supplemented with 1% L-glutamine, 100 U/mL penicillin/streptomycin and 10% FBS. 3T3-L1 were cultured in DMEM high glucose supplemented with 1% L-glutamine, 100 U/mL penicillin/streptomycin and 10% Bovine Calf Serum (BCS). Cells were maintained at 37 °C in a humidified atmosphere of 95% air and 5% CO_2_ and periodically screened for contamination [24,25].

#### 2.8.2. Cell Viability

Cells were grown in complete medium, and, before being treated, they were starved in serum-free medium for 24 h for the cell-cycle synchronization. Cells were then treated with increasing concentrations (from 0.001 to 100 μg/mL) of each extract dissolved in DMSO for 72 h. Cell viability was assessed using the 3-(4,5-dimethylthiazol- 2-yl)-2,5-diphenyltetrazolium bromide reagent (MTT), according to the manufacturer’s protocol (Sigma–Aldrich, Milan, Italy), as previously described [26]. For each sample, mean absorbance, measured at 570 nm, was expressed as a percentage of the control and plotted versus drug concentration to determine the IC_50_ values for each cell line, using GraphPad Prism 9 software (GraphPad Inc., San Diego, CA, USA). Mean values and standard deviations (SD) of three independent experiments carried out in triplicate and are shown.

#### 2.8.3. TUNEL Assay

TUNEL assay was used to assess apoptosis, following the guidelines of the manufacturer (CFQ488A TUNEL Assay Apoptosis Detection Kit, Biotium, Hayward, CA, USA), as previously described [27]. Cells were observed and imaged under a fluorescence microscope (Leica DM6000; 20× magnification) with excitation/emission wavelength of 490/515 nm (CF^TM^488A) or 350/460 nm (DAPI). Images are representative field of three separate experiments.

#### 2.8.4. Wound-Healing Assay

Cells were plated on 12-well plates and cultured in full medium to produce confluent monolayers. Cells were then wounded in a line and incubated with each extract at its IC_50_ value, as already described [28]. Images at time zero (t = 0 h) were acquired to record the initial area of the wound, and the recovery of the wounded monolayer due to cell migration toward the scratched area was estimated at 96 h (t = 96h). Images were acquired using an inverted microscope equipped with digital camera (Leica DM6000, 5×). The migration of cells toward the wounds was expressed as percentage of wound closure [Equation (1)]:

Wound closure % = [A(t = 0 h) − A(t = 96 h)]/A(t = 0 h)(1)

Vehicle-treated cells were used as a control. The collected images were analyzed with Leica Application Suite X (LAS X) software and the wound areas were measured using ImageJ. Representative images are shown. Each experiment was performed in triplicate.

#### 2.8.5. Immunofluorescence

For immunocytochemistry, cells were processed as already reported [28,29]. The primary mouse antibody against E-cadherin, N-cadherin, vimentin, VEGF, actin and tubulin were purchased from Santa Cruz Biotechnology Inc. (Santa Cruz, CA, USA). The secondary antibodies Alexa Fluor^®^ 568 conjugate goat anti-mouse was acquired from Thermo Fisher Scientific (Waltham, MA, USA). Nuclei were stained using DAPI 0.2 µg/mL (Sigma). Fluorescence was detected by using a fluorescence microscopy (Leica DM6000). All the images, processed with LAS-X software, are representative fields of three independent experiments.

#### 2.8.6. Detection of Intracellular Reactive Oxygen Species (ROS)

Human embryonic kidney Hek-293 cells were grown in 48-well plates and, after 24 h, were treated with the three extracts at a concentration of 0.1 μg/mL for 24 h and then we induced ROS production with menadione at 20 μM for 15 min. After treatment, cells were further processed using 2′-7′-Dichlorofluorescein diacetate (DCF-DA) as previously reported [30]. Finally, cells were observed and imaged under a fluorescence microscope, Leica Biosystems, Milan (MI), Italy (Leica DM6000; 20× magnification) with excitation/emission wavelength maxima of 490/515 nm (DCF) or 350/460 nm (DAPI, used for nuclei staining). Images are representative of three independent experiments. The ROS quantification in treated cells, shown as green fluorescence, was quantified using ImageJ.

#### 2.8.7. Statistical Analysis

Statistical analyses were performed by GraphPad Prism 8.0.2 software and tested for the different extracts using the one-way analysis of ANOVA followed by Tukey’s multiple range test, establishing the significance at *p* values < 0.05 (*), *p* < 0.01 (**), *p* < 0.001 (***), and *p* < 0.0001 (****). All the values were mean ( ± SD) of three experiments carried out in triplicate.

## 3. Results

### 3.1. Recovery of Extracts from A. cherimola Leaves

The recoveries and yields of the ethanolic (ACE), methanolic (ACM) and aqueous (ACW) extracts from the *A. cherimola* leaves were reported in Table 1. Data highlighted that water and methanol had a greater extraction efficacy (yield 17%) than ethanol (yield 11%).

### 3.2. Determination of Total Phenolic (TPC) and Flavonoid Content (TFC)

Polyphenols are phytochemicals distributed in all parts of the plant, including the leaves, which contribute significantly to the biological activity of plant extracts. The total phenolic content for each extract was reported in Table 1.

The highest phenolic content was found in the ACE (7.1 ± 0.1 µg GAE g^−1^) followed by the ACM (6.2 ± 0.2 µg GAE g^−1^). The ACW, although recovered in a higher amount than the others, has the lowest content of phenols (2.9 ± 0.2 µg GAE g^−1^), probably because it was richer in saponins than polyphenols. Ethanol and methanol had already extracted about 80% of the phenols present in the leaves. The TPC values of the three extracts were statistically different (**** *p* < 0.0001).

The TFC in the extracts of the *A. cherimola* leaves showed the same trend as the phenolic content (Table 1).

The flavonoid content in the ACE and ACM with 6.4 ± 0.1 and 5.8 ± 0.1 µg QE g^−1^, respectively, was significantly different (** *p* < 0.01). The flavonoid content in the ACW was found to be 0.9 ± 0.2 µg QE g^−1^, resulting much lower than that of the other two extracts (**** *p* < 0.0001). The ethanolic extract, even if recovered with the lowest yield, represented the one richest in bioactive compounds.

### 3.3. Determination of the Radical Scavenging Activity

Four concentrations ranging from 1.0 to 0.01 mg ml^−1^ of ACM, ACE and ACW were tested for their antioxidant activity in two different in vitro models, DPPH and ABTS assays, in order to establish a correlation with the phenolic content.

**Figure 1:**
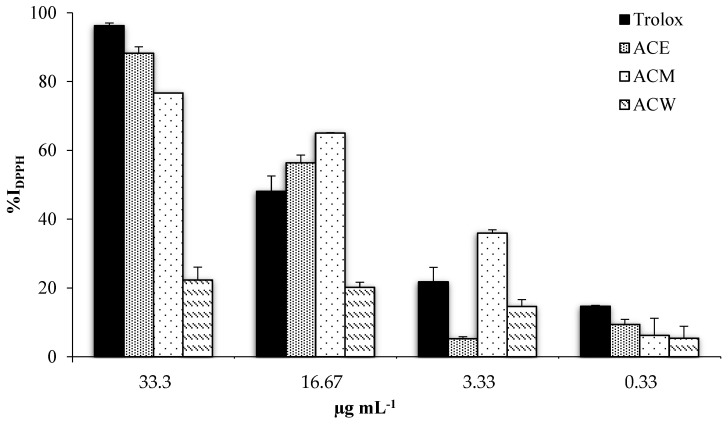
Graphical representation of DPPH inhibition percentage of the extracts from *A. cherimola* leaves.

**Figure 2:**
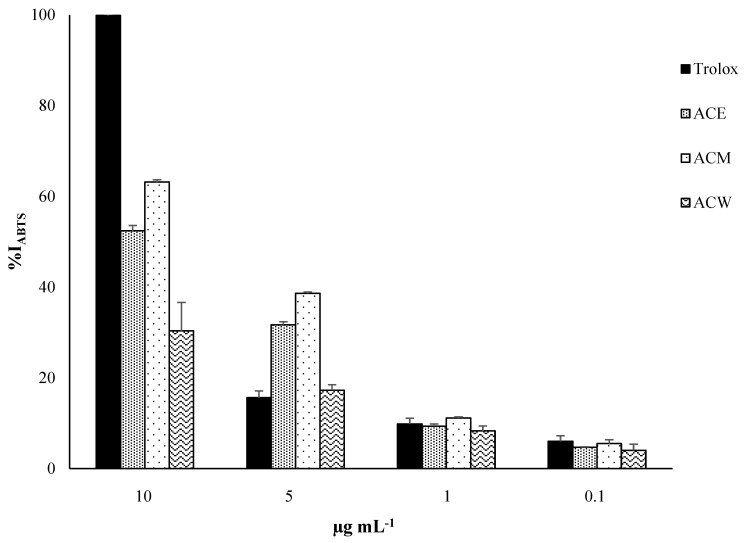
Graphical representation of ABTS inhibition percentage of the extracts from *Annona cherimola* leaves.

Both the DPPH and ABTS•^+^ radicals were scavenged by the three extracts in a concentration-dependent manner in both assays (Figure 1 and Figure 2). From the results, it was evident that the ACW exhibits the lowest antioxidant activity even at the highest concentration (22.3 ± 3.8 at 33.33 µg mL^−1^), in accordance with its total phenolic and flavonoid content, which represent notoriously antioxidant compounds.

The ACE, at the highest concentration, showed the highest inhibition percentage (88.3 ± 1.8) followed by the ACM at the same concentration (76.67 ± 0.1), which is more effective at lower concentrations (16.67 and 3.33 µg mL^−^^1^) in inhibiting the DPPH radical (65. 1 ± 1.1 and 33.3 ± 1.2, respectively) than the ethanolic one (58.9 ± 2.0 at 16.67 µg mL^−1^ and 9.4 ± 0.6 at 3.33 µg mL^−1^). Indeed, at 16.67 µg mL^−1^, both ACE and ACM inhibit the radical DPPH in a higher percentage than Trolox at the same concentration (48.1 ± 0.5). The EC_50_ values of the ethanolic, methanolic and aqueous extracts were found to be 11.2 ± 1.1, 8.2 ± 0.9 and 88.9 ± 1. 9 µg mL^−1^, respectively, indicating that methanolic extract is the most efficient in inhibiting the radical DPPH by 50% and comparable to Trolox (10.2 ± 1.0 µg mL^−1^). The TEAC values of the ACE, at concentrations of 33.3 and 16.3 µg mL^−1^, were 84.02 ± 0.7 and 48.34 ± 0.5 µmol Trolox equivalents per 100 g of dry extract, respectively, while the corresponding TEAC values for the ACM were 69.94 ± 0.1 and 55.76 ± 0.5 µmol Trolox equivalents per 100 g of dry extract (Table 2).

The results obtained from the ABTS assay highlighted that the extracts were less effective in inhibiting the cation radical ABTS•^+^ than the DPPH radical.

The results highlighted that the inhibition percentages at the highest concentration (10 µg mL^−1^) of the ACE (52.4 ± 1.1), ACM (63.1 ± 0.5) and ACW (30.3 ± 1.3) compared to that of Trolox (99.8 ± 0.2) was reduced by 50, 40 and 70%, respectively. At the concentration of 5 µg mL^−1^, the inhibition capacity of the ACE and ACM was twice (31.7 ± 0.7) and 2.5 times (38.6 ± 0.3) that of the Trolox one (15.6 ± 1.4), respectively. Water showed a scavenging activity comparable to the control one (17.2 ± 1.2). At a concentration of 1 µg mL^−1^, the inhibition percentage ranged from 8.3 ± 1.1 for the water to 11.1 ± 0.2 for the methanol. The ethanol exhibited a percentage of inhibition (9.3 ± 0.5) similar to the Trolox (9.8 ± 1.3) at the same concentration. At lower concentrations, the radical scavenging capacities of the extracts were slightly lower than Trolox. The EC_50_ values of the ACE, ACM and ACW were found to be 9.8 ± 0.1, 6.9 ± 0.8 and 22.7 ± 1.3 μg mL^−1^, respectively, showing that the methanolic extract possessed a good antioxidant capacity if compared to the Trolox one (EC_50_ equal to 5.7 ± 0.7 μg mL^−1^).

The TEAC values of the ethanolic extract, at concentrations of 10 and 5 µg mL^−1^, were 2.12 ± 0.3 and 1.13 ± 0.1 µmol Trolox equivalents per 100 g of dry extract, respectively, while the corresponding TEAC values for the methanolic extract were 2.53 ± 0.1 and 1.54 ± 0.2 µmol Trolox equivalents per 100 g of dry extract (Table 2).

### 3.4. Identification and Quantification of Phenolic Compounds by HPLC-DAD

In the ACM and ACE, five compounds were identified among the standards, while only two of them were present in the ACW (Table 3). The concentration of the identified compounds in each extract was determined from the corresponding calibration curves (gallic acid, y = 34,153x – 57,204, r^2^ = 0.992; chlorogenic acid, y = 529,633x – 174,104, r^2^ = 0.999; *p*-coumaric acid, y = 745,916x + 77,200, r^2^ = 1, ferulic acid, y = 771,161x – 236,647, r^2^ = 0.997; quercetin, y = 763,227x – 208,219, r^2^ = 0.998). The main compound in all three extracts was gallic acid followed by chlorogenic acid; the first one was contained in higher amounts in the ACM (75.7 ± 2.3 mg 100 g ^−1^) than in the ACE and ACW (60.0 ± 2.7 mg 100 g ^−1^ and 51.3 ± 1.9 mg 100 g ^−1^, respectively). The gallic and chlorogenic acids constituted 61% of the total weight of the identified phenolics in the ACE. Quercetin and ferulic acid were found in the same concentrations in both the ACM and ACE (31 and 36% of the total weight of the identified compounds, respectively).

The identified phenolic acids in the Cherimoya leaf extracts privilege the comprehension of their antioxidant capacities and pharmacological aspects [31]. Gallic acid showed better antioxidant capacity than the vitamin C one in both the DPPH and ABTS assays [32]. The high concentration of gallic acid in the methanolic extract was in accordance with its prevalent antioxidant capacity compared with the other extracts.

## 4. Biology

### 4.1. Anticancer Activity on Melanoma Cells

The cytotoxic effects of the *A. cherimola* leaf extracts (AC) were determined against two human melanoma cell lines, A2058, derived from a metastatic site, and malignant melanoma Sk-Mel-28, using an MTT assay. As a normal cell line, we employed the embryonal mouse fibroblasts 3T3-L1. The IC_50_ values, determined after 72 h of treatment at different concentrations (from 0.001 to 100 μg/mL, see Materials and Methods for details) of each of the AC extracts, are reported in Table 4. As a reference molecule, we used a well-known Vinca alkaloid, which is Vinblastine, already used in many studies [33,34]. As evidenced in Table 4, each AC extract was very active in diminishing the melanoma cells’ viability. Particularly, the major impact was observed for the metastatic cell lines A2058 for each AC extract, but a relevant anticancer activity was recorded also for the Sk-Mel-28 cells. For the A2058 cells, the extract with the higher activity was the ACE, with an IC_50_ of 5.6 × 10^−3^ ± 0.8 μg/mL, followed by the ACM and ACW, with IC_50_ values of 1.3 × 10^−2^ ± 0.6 and 2.4 ± 0.9 μg/mL, respectively. Regarding the Sk-Mel-28 cells, we calculated the following values, 0.12 ± 1.0, 0.19 ± 0.5 and 23.3 ± 0.8 μg/mL, for the ACE, ACM and ACW, respectively. It is important to highlight that Vinblastine, used in comparison with our extracts, exerted a stronger anticancer activity against the A2058 melanoma cell lines (IC_50_ equal to 8.6 × 10^−4^ ± 0.8 μg/mL). As well for the Sk-Mel-28 cells, Vinblastine activity (IC_50_ equal to 15.3 ± 0.7 μg/mL) was higher than the ACW but was impressively lower than those from the ACE and ACM (see Table 4). These properties come together with a high difference in affecting the normal 3T3-L1 cells’ viability. Indeed, Vinblastine produced a remarkable cytotoxic effect against the 3T3-L1 cells (IC_50_ of 1.6 × 10^−4^ ± 0.7 μg/mL), sensibly higher than in the two melanoma cell lines, indicating a lack of selectivity. Contrarily, all the AC extracts affected the 3T3-L1 cells’ viability to a much lesser extent, and as an example, the ACE activity against the A2058 melanoma cells was almost 5 × 10^−3^ superior to that exerted against the 3T3-L1 cells. In conclusion, we proved that the cytotoxic profile of all the AC extracts was better than that of Vinblastine, pointing out the attention on the higher selectivity among cancer and normal cells.

### 4.2. A. cherimola Mill. Leaf Extracts induce Apoptosis in A2058 Melanoma Cells and Reduce Their Migration

**Figure 3:**
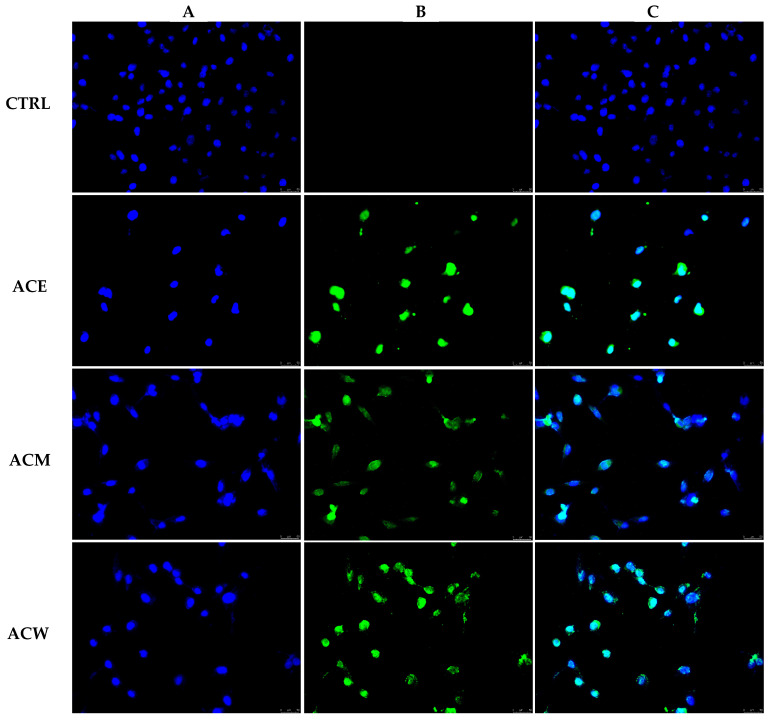
Apoptosis detection by TUNEL assay on A2058 cells treated with ethanolic, methanolic and aqueous *A. cherimola* Mill. leaf extracts (ACE, ACM and ACW) at their IC_50_ values or with vehicle (DMSO, CTRL) for 24 h. Cells were then subjected to the TUNEL procedure (as reported in Materials and Methods) and imaged under an inverted fluorescence microscope at 20× magnification. (**A**) DAPI (λ_ex/em_ = 350/460 nm); (**B**) CF^TM^488A (λ_ex/em_ = 490/515 nm); (**C**) overlay channel. Images are representative fields of three independent experiments.

In order to understand if the observed diminution in cell viability was due to a killing effect, we exposed the A2058 melanoma cells for 24 h to ACW, ACE and ACM at concentrations equal to their IC_50_ values. Then, we evaluated the DNA damage (a characteristic hallmark of apoptosis) and the occurring cell death by performing the TUNEL assay. As a negative control, we exposed the A2058 melanoma cells, at the same experimental conditions, to the vehicle used to solubilize the AC extracts (DMSO). The cells were then processed, as described in the Section 2, and observed under a fluorescence microscope. As is visible in Figure 3, the damaged and fragmented DNA, indicated by a green nuclear fluorescence, is present only in the nuclei of the A2058 melanoma cells treated with all the extracts (Figure 3, Panels B, ACE, ACM and ACW), whereas it is totally absent in the nuclei of the vehicle-treated cells (Figure 3, Panel B, CTRL). The overlay among the green (fragmented DNA) and blue (cell nucleus) fluorescence is shown in the panels in column C. Thus, we can conclude that the exposure, for only 24 h, to all the AC extracts is able to trigger the A2058 melanoma cells’ death by apoptosis.

**Figure 4:**
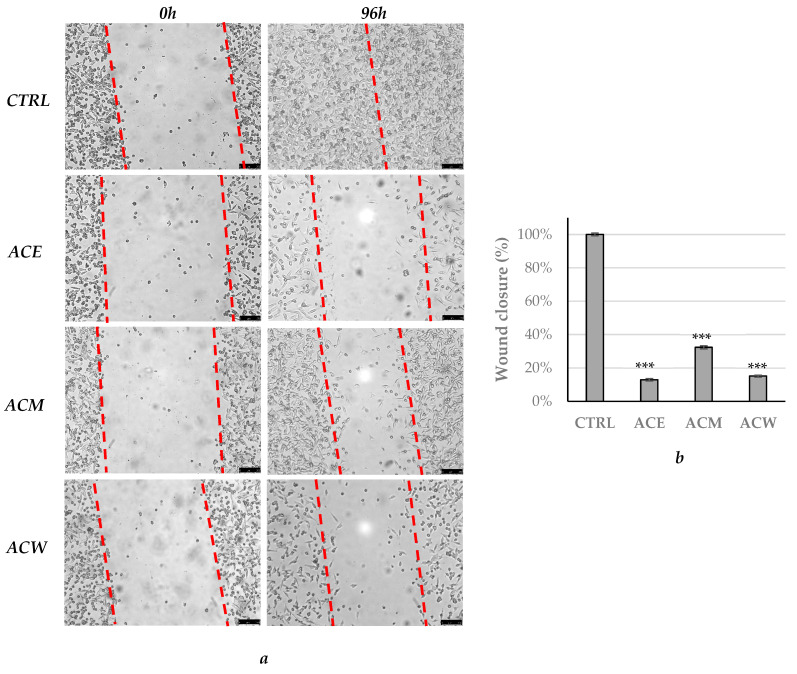
(**a**) Wound-healing assay conducted on A2058 cells treated with ethanolic, methanolic and aqueous *A. cherimola* Mill. leaf extracts (ACE, ACM and ACW) at their IC_50_ values or with vehicle (DMSO, CTRL). Wound closure was monitored at 0 and 96 h using an inverted microscope (5× magnification). Dotted red lines define the areas that lack cells. (**b**) The percentage of wound closure was calculated using ImageJ, as reported in Materials and Methods, in order to estimate the wound-healing effect. *** *p* < 0.001, ACE, ACM and ACW vs. CTRL.

Next, we evaluated the ability of the AC extracts to regulate the potential migration of the A2058 melanoma cells by the means of an easy, low-cost and well-developed in vitro method, namely the wound-healing assay. For this purpose, the cells were plated and allowed to form a monolayer, then scratched to form a wound and exposed to the AC extracts at their IC_50_ values (see Table 4), taking care to monitor the cells behavior each 24 h in order to find the final time-point to which the wound has closed in the vehicle-treated cells (DMSO, positive control). These observations allowed to estimate that the closure was complete (100%) after 96 h, under DMSO treatment and the adopted experimental conditions. The A2058 melanoma cells are highly aggressive, invasive and tend to give metastases and thus represent a good model to evaluate the potential of our extract to inhibit these processes, in vitro. As reported in Figure 4, the ACE produced the best effect, impeding the complete wound closure, followed by the ACW and ACM, with percentages of about 13, 15 and 32% (Figure 4b), respectively. It is noticeable that the ACE possesses the highest activity, diminishing both the viability and migration of the A2058 melanoma cells, whereas the ACW has a lesser impact on cell viability than the ACM (see Table 4) but a higher efficacy in blocking cell migration (Figure 4). Summing up, our data indicate that ACE possesses the best activity toward the A2058 melanoma cells; thus, it was chosen for further investigations.

### 4.3. ACE Regulates E- and N-Cadherin, Vimentin and VEGF Expression in A2058 Melanoma Cells

**Figure 5:**
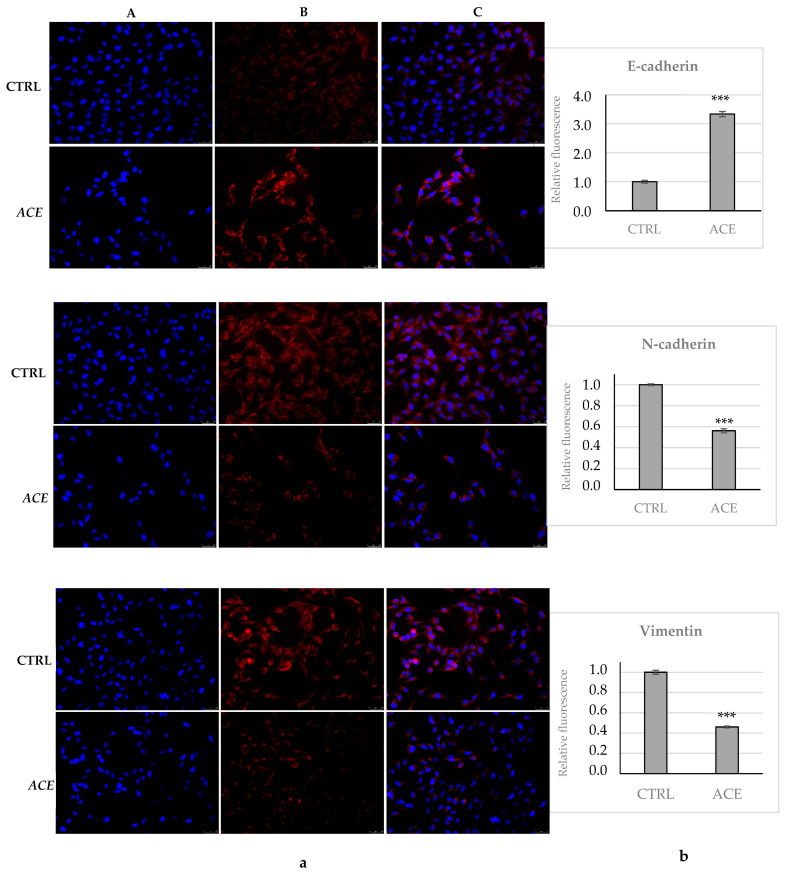
Immunofluorescence analysis of E-cadherin, N-cadherin and Vimentin. (**a**) A2058 cells were treated with ethanolic *A. cherimola* Mill. leaf extracts (ACE) at its IC_50_ value or with vehicle (DMSO, CTRL). Images were acquired at 20× magnification. ACE exposure increased E-cadherin expression and reduced N-cadherin and vimentin expression in A2058 cells. (**A**) DAPI (λ_ex/em_ = 350/460 nm); (**B**) Alexa Fluor^®^568 (λ_ex/em_ = 644/665 nm); (**C**) overlay channel. Images are representative fields of three independent experiments. (**b**) Fluorescence quantification was obtained using ImageJ. *** *p* < 0.001, ACE vs. CTRL.

For a better understanding of the ACE action in A2058 cell migration and invasion, we decided to investigate the expression levels of the three proteins mainly involved in the epithelial-to-mesenchymal transition (EMT) process, namely E- and N-cadherin and vimentin. Briefly, the cells were exposed for 24 h to the ACE (concentration equal to its IC_50_ value) then processed and subjected to immunofluorescence analysis, using specific antibodies against E- and N-cadherin and vimentin. As a control, vehicle-treated cells were used. First, a net increase in E-cadherin expression was observed under the ACE treatment, almost 3.5-folds higher than in the vehicle-treated cells (Figure 5a,b, E-cadherin), whereas the expression of N-cadherin was around half (Figure 5a,b, N-cadherin). In agreement with these outcomes, the vimentin expression was also found to be half, under ACE treatment, with respect to the control cells (only vehicle), as visible in Figure 5a,b, Vimentin. Thus, all these observations indicate that ACE exposure is able to impede the A2058 melanoma cells’ invasiveness potential by regulating the expression levels of three of the major actors playing a role in the EMT.

**Figure 6:**
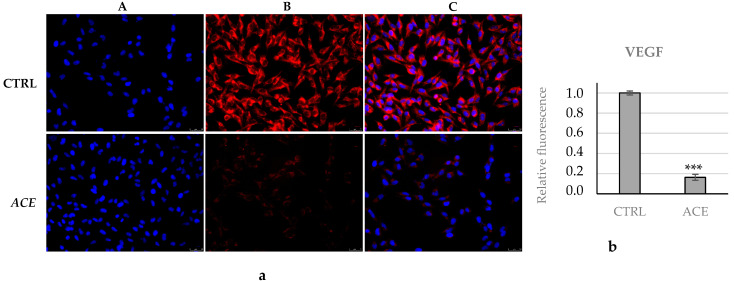
Immunofluorescence analysis of VEGF. (**a**) A2058 cells were treated with ethanolic *A. cherimola* Mill. leaf extracts (ACE) at its IC_50_ value or with vehicle (DMSO, CTRL)**.** ACE exposure reduced VEGF expression in A2058 cells. Images were acquired at 20× magnification. (**A**) DAPI (λ_ex/em_ = 350/460 nm); (**B**) Alexa Fluor^®^568 (λ_ex/em_ = 644/665 nm); (**C**) overlay channel. Images are representative fields of three independent experiments. (**b**) Fluorescence quantification was obtained using ImageJ. *** *p* < 0.001, ACE vs. CTRL.

Finally, it is noteworthy that tumor progression and metastasis onset need new blood vessels (angiogenesis), which provide the required resources and allow the dissemination around the body [35]. Among many factors, VEGF is undoubtedly one of the most studied regulators playing a major role in angiogenesis; thus, we evaluated its expression in our model. Our outcomes (Figure 6a,b) evidenced a significant diminution of VEGF expression in A2058 cells, under ACE exposure, of about five times compared to the vehicle-treated cells. Considering that cancer cells may release VEGF to initiate angiogenesis and enhance the blood supply to the tumor, allowing the uncontrolled growth and, unfortunately, the dissemination of cancer cells, the ability of our extract to diminish this phenomenon is, in agreement with the above-mentioned data, of great importance in blocking A2058 cells’ invasiveness.

### 4.4. ACE Perturbs the A2058 Cells Cytoskeleton Structure

**Figure 7:**
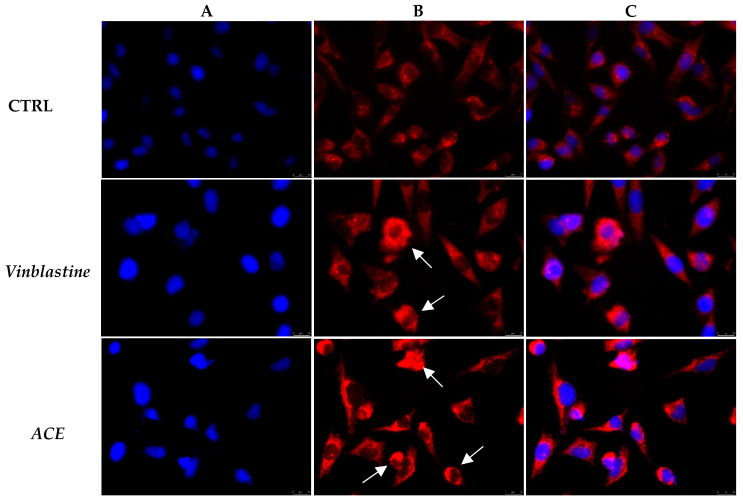
β-tubulin immunofluorescence analysis. A2058 cells were treated with ethanolic *A. cherimola* Mill. leaf extracts (ACE) at its IC_50_ value, with Vinblastine at 1 µM or with vehicle (DMSO, CTRL). Images were acquired at 40× magnification. ACE affected the normal tubulin assembly, similarly to Vinblastine. (**A**) DAPI (λ_ex/em_ = 350/460 nm); (**B**) Alexa Fluor^®^568 (λ_ex/em_ = 644/665 nm); (**C**) overlay channel. Images are representative fields of three independent experiments.

In order to investigate whether ACE may act on the microtubule system, we performed immunofluorescence assays on A2058 cells exposed to the ACE (at its IC_50_ value) or vehicle for 24 h, using the Vinblastine (1 µM) as the positive control. Our data indicated that the vehicle-treated cells show an even distribution and organization of the microtubules (Figure 7, panels B, CTRL), whereas under Vinblastine treatment, the microtubules appear thicker and brighter, accumulating in disorganized structures (Figure 7, panels B, Vinblastine, white arrows). Moreover, the presence of some bi- or tri-nucleated cells suggests an abnormal mitotic arrest that is a consequence of the altered microtubule assembly. A Vinblastine-like effect has been evidenced in ACE-treated cells as well (Figure 7 panel B, ACE, white arrows), indicating that the extract affects the normal tubulin assembly.

**Figure 8:**
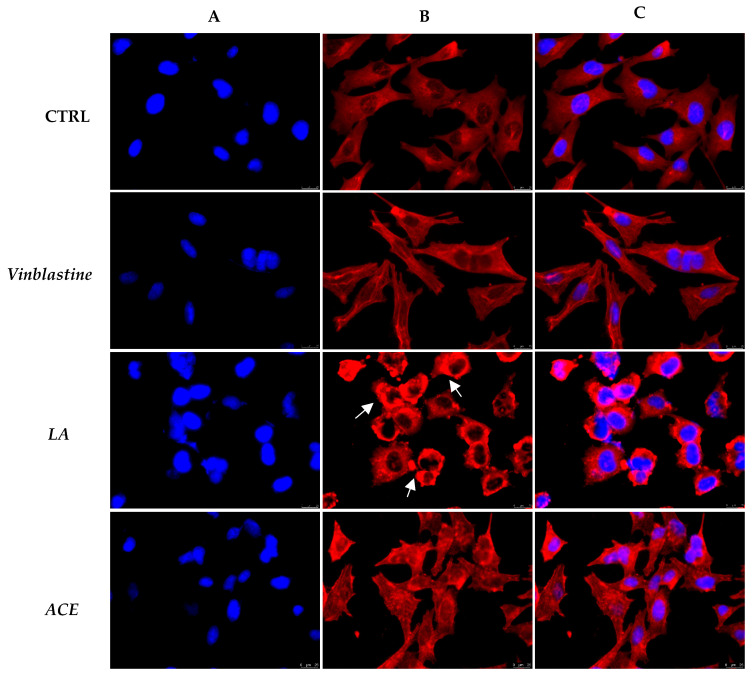
β-actin immunofluorescence analysis. A2058 cells were treated with ethanolic *A. cherimola* Mill. leaf extracts (ACE) at its IC_50_ value, with Vinblastine at 1 µM, with LA at 1 µM or with vehicle (DMSO, CTRL)**.** Images were acquired at 40× magnification. ACE induced a disorganization of the actin system with the presence of actin cables at the cell periphery, in a similar fashion to Vinblastine. (**A**) DAPI (λ_ex/em_ = 350/460 nm); (**B**) Alexa Fluor^®^568 (λ_ex/em_ = 644/665 nm); (**C**) overlay channel. Images are representative fields of three independent experiments.

Next, we wondered if the other major cytoskeletal component, namely actin, could be interested by ACE exposure. In this case, we used Latrunculin A (LA) as the reference molecule, a well-known natural compound selectively targeting the actin, at a concentration of 1 µM, under the same experimental conditions, and Vinblastine as well. As visible in Figure 8 (panel B, CTRL) in the negative control (only DMSO), the actin filaments are fairly distributed in the cytoplasm. Contrarily, the exposure to the LA induced a clear compacting of the actin system, mostly around the cell nuclei along with the presence of many dot-like structures (Figure 8, panel B, LA, see white arrows). Under Vinblastine treatment, the actin system is changed with respect to the DMSO-treated cells, and prominent and straight actin cables are present at the cell periphery (Figure 8, Panel B, Vinblastine). However, a net difference in the actin arrangement between the A2058 cells treated with LA or Vinblastine is noticeable. Under ACE exposure, the actin system appears not fairly distributed in the cell cytoplasm (Figure 8, panel B, ACE) and actin cables are present at the cell periphery. This tidiness is very similar to that produced by the Vinblastine treatment instead of that of the LA (Figure 8, panels B, Vinblastine and LA, respectively), leading to the deduction that ACE may act indirectly on the actin system. Indeed, the interference with the microtubules affects the normal actin organization, as happens under Vinblastine exposure.

### 4.5. Antioxidant Properties of AC Extracts in Human Embryonic Kidney Hek-293 Cells

**Figure 9:**
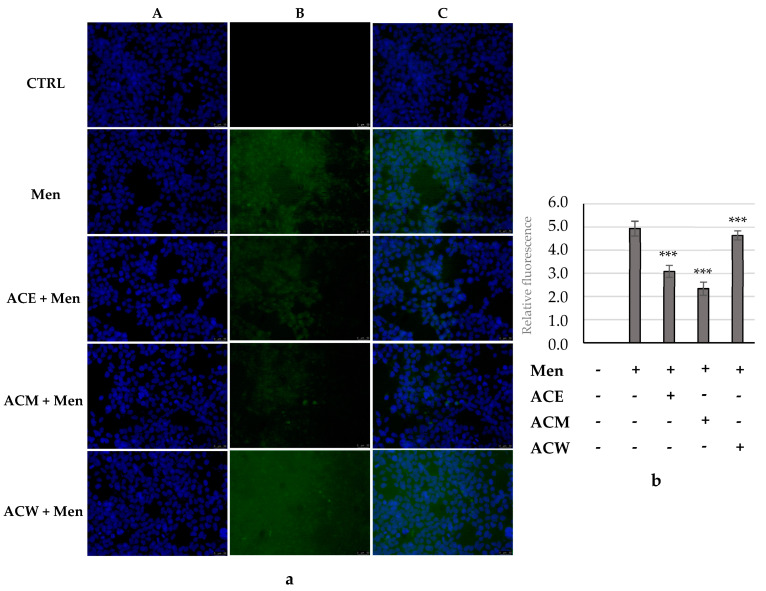
ROS scavenging activity. (**a**) Evaluation of ROS scavenging activity using DCF-DA (CF^TM^488A) in Hek293 treated with Men (used as ROS inducer) or co-treated with Men and each *A. cherimola* Mill. leaf extract (ACE, ACM and ACW) at a concentration of 0.1 µg/mL. Images were acquired at 20× magnification. ACE and ACM pre-treatment reduce ROS production induced by Men of about 2- and 2.5-folds, whereas ACW demonstrated a lower but significant ROS scavenger activity. (**A**) DAPI (λ_ex/em_ = 350/460 nm); (**B**) CF^TM^488A (λ_ex/em_ = 490/515 nm); (**C**) overlay channel. (**b**) Fluorescence quantification; *** *p <* 0.001, Men+ACE, Men + ACM and Men + ACW vs. Men.

Based on the effective antioxidant activity, TPC and TFC content, the three AC leaf extracts were investigated for their ability to regulate the production of intracellular ROS levels, particularly superoxide anion (O_2_^−•^) and hydrogen peroxide (H_2_O_2_), induced by menadione exposure (vitamin K3, Men). For this purpose, we treated the human embryonic kidney Hek-293 cells with the three extracts at a concentration of 0.1 μg/mL for 24 h and then we induced ROS production with menadione at 20 μM for 15 min. After that, the cells were processed as described in the Section 2. As evidenced in Figure 9a (panel B, Men), menadione treatment induces elevated ROS production that is lacking the vehicle-treated cells (Figure 9a, panel B, CTRL). The pre-treatment with all the AC leaf extracts is able to diminish the induced ROS production in Hek-293 cells in all cases, with some differences. Indeed, the quantification of the green fluorescence associated with the ROS intracellular levels indicated that the ACW produced a very limited, but significant, ROS reduction (Figure 9b, ACW + Men). The best antioxidant effects have been recorded under ACM and ACE exposure which have been able to reduce ROS levels about 2- and 2.5-folds, respectively (Figure 9b, ACM and ACE, respectively). It is worthy to note that the Hek-293 cells viability, under the concentration and conditions used for these assays, was over 90%, as resulted from a previous MTT assay (data not shown). These outcomes are in agreement with the previous antioxidant tests, TPC and TFC quantification, corroborating the proof of the high potential of ACM and ACE in preventing the oxidative stress.

## 5. Discussion

*A. cherimola* Mill., belonging to the *Annonaceae* family, is a traditional semi-evergreen plant commonly known as Cherimoya, which derives from the word ‘chirimuya’, meaning “cold seed”. It is distributed in the tropical or subtropical regions of America, Africa, Asia and the south of Europe, and its fruit is a concentration of nutraceuticals with different benefits for human health [36]. However, even the leaves have been used in traditional medicine to treat several conditions and they represent a great source of phytochemicals, among them flavonoids, tannins, alkaloids, phytosterols and terpenoids. *A. cherimola* Mill. found a favorable microclimate in Reggio Calabria (Italy), where it is successfully cultivated, and its fruit is used to prepare extraordinary and lovely dishes (ice creams, sorbets, jams and so on); unfortunately, the leaves are usually discarded during the fruit harvesting, contributing to the accumulation of agricultural waste to be disposed of. For this purpose, and considering their high pharmaceutical and nutraceutical potential, we decided to collect leaves from *Autoctona Calabrese* cultivar and obtain three extracts, namely ethanolic (ACE), methanolic (ACM) and aqueous (ACW), for which the phenolic profile, flavonoid content and antioxidant capacity have been investigated. The total phenolic (TPC) and flavonoid content (TFC) were higher in the ACE than in the other two extracts and, particularly, the gallic and chlorogenic acids were found in all the extracts, but in higher concentrations in the ACE and ACM than the ACW, whereas the quercetin, p-coumaric and ferulic acids were found only in the ACM and ACE. This composition reflects the antioxidant activities determined in the scavenging assays (DPPH and ABTS), where the ACE and ACM represent the most efficient antioxidant extracts. Furthermore, we also tested the antioxidant properties of the three extracts in a cell model, namely the human embryonic kidney Hek-293 cells, using Menadione as an oxidative stress inducer. Our outcomes showed that the ACE and ACM extracts were the most efficient in contrasting the induced oxidative stress, even though there was a little difference with respect to the previous DPPH and ABTS assays, where the ACE was the better antioxidant. This could be due to several reasons, for instance, the different ability to pass the cell membranes or the possible interaction with the intracellular redox enzymatic systems [37]. Next, we investigated the anticancer properties of the extracts using two human melanoma cell lines, A2058 and Sk-Mel-28, finding out that the ACE was the most active against the A2058 cells, with an IC_50_ equal to 5.6 × 10^−3^ ± 0.8 µg/mL, followed by the ACM and, to a lesser extent, the ACW. A similar effect has been observed for Sk-Mel-28 cells, together with a negligible cytotoxicity against the normal murine fibroblasts 3T3-L1. These results are very encouraging and are in agreement with some of the reported literature data [18,38] even though, to our best knowledge, we reported very low IC_50_ values and a selectivity between the melanoma and normal cells that makes these extracts worthy of further studies regarding the possible intracellular targets and mechanisms by which they exert the anticancer activity. Thus, we first evaluated the possible mechanism of melanoma cell death of all the extracts, observing that they induce apoptosis after only 24 h of exposure, as evidenced by the fluorescence associated with the fragmented DNA, not visible in the vehicle-treated cells, used as the control. As well, this observation is in agreement with other studies [18], even though we used A2058 melanoma cells and very low extract concentrations. The observed apoptotic death comes together with another important outcome, namely the ability to block the melanoma cell migration. By the means of a validated and simple method, i.e., the wound-healing assay, we demonstrated that all the extracts have been able to block the directional A2058 cells migration in vitro, the ACE and ACW being the best ones, whereas in the only vehicle-treated cells, the scratch has been completely closed. Taking into account these results, it is evident that ACE is the extract with the best antimelanoma activity; thus, we focused the next studies on this one and decided to check the status of three, among the most important, proteins involved in cancer cell migration and metastasis formation, namely E- and N-cadherin and vimentin. The literature data report that, during the EMT, the malignant cells can rearrange their cytoskeleton, lose the epithelial cell junction proteins such as, for instance, E-cadherin, and increase the expression of mesenchymal markers, such as N-cadherin and vimentin [39,40]. With this in mind, we performed IF studies on A2058 cells exposed to ACE for 24 h. As expected from our previous observations, the expression levels of the three considered proteins vary under ACE treatment with respect to the control (only vehicle). Particularly, we observed a rise of the E-cadherin expression, of about 3.5-folds, and a diminution of N-cadherin and vimentin expression, of about half, with respect to the control (vehicle-treated cells).

Melanoma is an aggressive cancer with a higher risk of metastatic spread and, commonly, the primary tumoral site of melanoma gives rise to new blood vessels, a phenomenon known as angiogenesis, necessary to provide nutrients for cancer cell survival, uncontrolled growth and progression [41]. The rise of pro-angiogenic factors is induced by many mechanisms, among them the production of VEGF that can exert paracrine and autocrine effects and contribute to the new vessels’ formation [42,43]. Thus, we evaluated the VEGF intracellular expression after ACE exposure, evidencing a net diminution of about 5 times with respect to vehicle-treated cells. Summing up, the ACE treatment clearly reduces melanoma cells viability and blocks the metastatic potential by positively influencing some characteristic markers of the EMT and angiogenesis. Last but not least, the cytoskeleton, a complex intracellular structure, vital for many cell processes, is strictly connected, as well, with the tumor progression and the EMT process. Among the numerous components, two major ones are tubulin and actin which have been targets of potent anti-cancer drugs, from natural sources or chemical synthesis [29,44,45,46]. The reorganization of the actin cytoskeleton is fundamental for the migration and invasion of cancer cells, and has recently attracted the interest of many research groups, while the microtubule network provides the driving force for cell migration [47]. Additionally, the microtubule system damage is responsible for apoptosis in cancer cells, and several compounds may cause microtubules depolymerization or increase tubulin polymerization, such as Vinblastine and Paclitaxel, respectively. Thus, we investigated, as well, the status of tubulin and actin in A2058 melanoma cells under ACE exposure, finding out that ACE induces a clear disorganization of the microtubule network, similarly to Vinblastine, used as reference molecule. Moreover, a perturbation of the actin cytoskeleton has also been noticed under ACE treatment, but this effect could be due not to a direct interaction of our extract with the actin but presumably as a consequence of the effect on microtubules. Indeed, the actin organization under ACE exposure is comparable to that obtained under Vinblastine treatment rather than that visible under LA treatment. As it is known, LA is a strong inhibitor of actin polymerization, being able to bind and sequester G-actin monomers, and a promoter of the depolymerization, favoring the subunits’ dissociation from the assembled filaments [48]. However, the microtubule perturbation strongly influences the actin cytoskeleton [49] and this explicates the different pattern observed under ACE exposure with respect to that of LA. In conclusion, ACE has been proved to possess antimelanoma and antioxidant properties, being able to prevent melanoma cell growth and migration through the regulation of the main proteins involved in EMT and angiogenesis, and perturbing the melanoma cells cytoskeleton organization. The presented outcomes provide new knowledge to be exploited for a desirable, smart future re-use of *A. cherimola* leaves, for instance, as a food supplement, consider the positive effects that could have on human or animal health and, at the same time, minimize agricultural waste and pollution.

## 6. Conclusions

Herein, we proposed a re-use of *A. cherimola* leaves, subjected to chemical extraction and investigation of the antioxidant and antimelanoma properties. Our studies revealed that the most active ethanol extract induces melanoma cell apoptosis and reduces the metastatic potential through the regulation of cell cytoskeleton dynamics, EMT and angiogenesis markers. The high antimelanoma activity and negligible impact on normal cell viability make this extract worthy of note, suggesting a smart recycling of an agricultural by-product.

## Figures and Tables

**Table 1:** Recovery, yield, TPC and TFC of the extracts from *A. cherimola* leaves.

Extract	Recovery (mg g^−1^)	Yield (%)	GAE (µg g^−1^_extract_)	QE (µg g^−1^_extract_)
**ACE**	111.2 ± 1.7	11.2 ± 0.17 ^b^	7.1 ± 0.1 ^a^	6.4 ± 0.1 ^a^
**ACM**	168.7 ± 2.3	16.9 ± 0.23 ^a^	6.2 ± 0.2 ^b^	5.8 ± 0.1 ^b^
**ACW**	169.0 ± 2.0	17.0 ± 0.2 ^a^	2.9 ± 0.2^c^	0.9 ± 0.2 ^c^

For each column, the statistical analysis, as measured by Tukey’s multiple range test, was indicated by the letters. Letter “a” denotes the highest content and different lowercase letters indicated significant differences. Yield: methanol and water vs. ethanol, *p* < 0.0001; QE: ethanol vs. methanol, *p* < 0.01; ethanol and methanol vs. water, *p* < 0.0001.

**Table 2:** Radical scavenging activity (ABTS and DPPH) of three extracts from *A. cherimola* leaves, expressed as µmol of Trolox equivalents per 100 g of dry extract.

	DPPH		ABTS	
Extracts	Concentrations (µg mL^−^^1^)			
	33.33	16.67	10	5
**ACE**	84.02 ± 0.7 ^a^	48.34 ± 0.4 ^a^	2.12 ± 0.3 ^a^	1.13 ± 0.1 ^a^
**ACM**	69.94 ± 0.1 ^b^	55.76 ± 0.5 ^b^	2.53 ± 0.1 ^a^	1.54 ± 0.2 ^a^
**ACW**	3.83 ± 0.2 ^c^	1.25 ± 0.3 ^c^	1.17 ± 0.2 ^b^	0.64 ± 0.1 ^b^

For each column, the statistical analysis, as measured by Tukey’s multiple range test, was indicated by the letters. Letter “a” denotes the highest content and different lowercase letters indicated significant differences. DPPH: *p* < 0.0001; ABTS: at 10 µg mL^−1^ ethanol vs. water, *p* < 0.01; methanol vs. water, *p* < 0.001; at 5 µg mL^−1^, methanol vs. water, *p* < 0.05.

**Table 3:** Content of phenolic compounds in the *A. cherimola* leaf extracts (mg 100 g^−1^ of dry extract).

	*Annona Cherimola Leaf Extracts*		
Standard	*ACE*	*ACM*	*ACW*
**Gallic acid**	60.0 ± 2.7	75.7 ± 2.3	51.3 ± 1.9
**Chlorogenic acid**	34.3 ± 1.1	38.1 ± 1.7	33.7 ± 0.8
***p*-coumaric acid**	10.3 ± 0.7	14.7 ± 1.8	n.d.
**Ferulic acid**	30.8 ± 2.3	30.8 ± 1.5	n.d.
**Quercetin**	27.3 ± 1.8	27.2 ± 0.5	n.d.

**Table 4:** IC_50_ values of ethanolic, methanolic and aqueous *Annona cherimola* Mill. leaf extracts (ACE, ACM and ACW, respectively) expressed in µg/mL.

IC_50_ (µg/mL)			
Extracts	A2058	Sk-Mel-28	3T3-L1
**ACE**	5.6 × 10^−3^ ± 0.8	0.12 ± 1.0	29.7 ± 0.6
**ACM**	1.3 × 10^−2^ ± 0.6	0.19 ± 0.5	3.6 ± 1.0
**ACW**	2.4 ± 0.9	23.3 ± 0.8	60.9 ± 1.1
**Vinblastine**	8.6 × 10^−4^ ± 0.8	15.3 ± 0.7	1.6 × 10^−4^ ± 0.7

## Data Availability

Data is contained within the article.

## Abbreviations

AC (*Annona cherimola* Mill. leaf extract); ACE (*Annona cherimola* Mill. leaf ethanolic extract); ACM (*Annona cherimola* Mill. leaf methanolic extract); ACW (*Annona cherimola* Mill. leaf aqueous extract); GA (gallic acid); QE (Quercetin); TPC (Total phenolic compound); TFC (Total flavonoid compound); TE (Trolox equivalent); DPPH (2,2-diphenyl-1-picrylhydrazyl radical); ABTS (2,2′-azino-bis (3-ethylbenzothiazoline-6-sulphonic acid) diammonium salt); AlCl_3_ (aluminium chloride); MTT (3-(4,5-dimethylthiazol- 2-yl)-2,5-diphenyltetrazolium bromide reagent), DCF-DA (menadione, 2’-7’-dichlorofluorescein diacetate); FBS (fetal bovine serum); EMT (epithelial-to-mesenchymal transition); DAPI (4′,6-diamidino-2-phenylindole); ROS (reactive oxygen species); Men (Menadione); DMEM (Dulbecco’s modified Eagle’s medium).
